# 3D myocardial *T*_1_ mapping using saturation recovery

**DOI:** 10.1002/jmri.25575

**Published:** 2017-02-02

**Authors:** Giovanna Nordio, Markus Henningsson, Amedeo Chiribiri, Adriana D.M. Villa, Torben Schneider, René M. Botnar

**Affiliations:** ^1^ Division of Imaging Science and Biomedical Engineering King's College London London UK; ^2^ Philips Health Systems London UK; ^3^ Wellcome Trust and EPSRC Medical Engineering Center, King's College London UK; ^4^ BHF Centre of Excellence, King's College London UK; ^5^ NIHR Biomedical Research Centre, King's College London; ^6^ Pontificia Universidad Católica de Chile, Escuela de Ingeniería Santiago, Chile

**Keywords:** *T*_1_ mapping, saturation‐recovery, MRI, accuracy, precision

## Abstract

**Purpose:**

To propose a 3D quantitative high‐resolution *T*_1_ mapping technique, called 3D SASHA (saturation‐recovery single‐shot acquisition), which combines a saturation recovery pulse with 1D‐navigator‐based‐respiratory motion compensation to acquire the whole volume of the heart in free breathing. The sequence was tested and validated both in a *T*_1_ phantom and in healthy subjects.

**Materials and Methods:**

The 3D SASHA method was implemented on a 1.5T scanner. A diaphragmatic navigator was used to allow free‐breathing acquisition and the images were acquired with a resolution of 1.4 × 1.4 × 8 mm^3^. For assessment of accuracy and precision the sequence was compared with the reference gold‐standard inversion‐recovery spin echo (IRSE) pulse sequence in a *T*_1_ phantom, while for the in vivo studies (10 healthy volunteers) 3D SASHA was compared with the clinically used 2D MOLLI (3‐3‐5) and 2D SASHA protocols.

**Results:**

There was good agreement between the *T*_1_ values measured in a *T*_1_ phantom with 3D SASHA and the reference IRSE pulse sequences (1111.6 ± 31 msec vs. 1123.6 ± 8 msec, *P* = 0.9947). Mean and standard deviation of the myocardial *T*_1_ values in healthy subjects measured with 2D MOLLI, 2D SASHA, and 3D SASHA sequences were 881 ± 40 msec, 1181.3 ± 32 msec, and 1153.6 ± 28 msec respectively.

**Conclusion:**

The proposed 3D SASHA sequence allows for high‐resolution free‐breathing whole‐heart *T*_1_‐mapping with *T*_1_ values in good agreement with the 2D SASHA and improved precision.

**Level of Evidence:** 2

**Technical Efficacy:** Stage 1

J. MAGN. RESON. IMAGING 2017;46:218–227

Myocardial fibrosis is a common manifestation for several cardiomyopathies and it is one of the main predictors of future cardiac outcome.^1^ Quantitative *T*_1_ mapping is a noninvasive tissue characterization technique that allows to differentiate between healthy and diseased tissues, based on the different environment of their water molecules.^2^ In recent years myocardial *T*_1_ mapping techniques have been widely used to visualize both local and diffuse fibrosis.^3^, ^4^ *T*_1_‐mapping compares favorably to the late gadolinium enhancement (LGE) technique both in terms of quantification/staging of disease and fibrosis detection, as LGE can only detect the presence of focal fibrosis in the heart.^2^ *T*_1_ mapping sequences acquire multiple images with variable *T*_1_ weighting and uses pixel‐wise fitting of the imaging data to an exponential signal recovery model of the longitudinal relaxation to generate a *T*_1_ map. *T*_1_ mapping is often performed before and after the administration of a contrast agent to quantify the extracellular volume fraction.^5^ Different techniques have been proposed for *T*_1_ mapping using either inversion‐recovery,^6^ saturation‐recovery,^7^ or a combination of the two.^8^ The modified Look–Locker inversion‐recovery (MOLLI) technique uses a 180° pulse to invert the longitudinal magnetization and a number of single‐shot images are acquired during the mid‐diastolic rest period at different inversion times.^6^ A number of “pause” cardiac cycles are typically performed, in which no images are acquired, to allow the longitudinal magnetization to fully recover between successive inversion pulses. This technique provides precise *T*_1_ maps with high reproducibility; however, the quantification accuracy is affected by the perturbation of the longitudinal signal recovery due to the repeated image acquisitions, causing a general underestimation of myocardial *T*_1_.^9^ The MOLLI technique is highly dependent on heart rate and this can severely affect the accuracy, as higher heart rates may not allow the longitudinal magnetization to fully recover to the initial state. A different solution has been proposed, which uses a saturation‐recovery pulse, called the saturation‐recovery single‐shot acquisition (SASHA) imaging sequence.^7^ With this technique, one single‐shot image is acquired after the application of a saturation pulse, typically in mid‐diastole. This is repeated in subsequent heartbeats with varying time delays after the saturation pulse, thereby providing images with varying *T*_1_ weighting. Thanks to the use of a saturation pulse, which removes any spin history, the SASHA sequence is less susceptible to *T*_1_ errors caused by heart rate variations^10^ and there is no requirement for pause cycles between image acquisition, making the scan efficiency higher compared to MOLLI. However, there is a loss in precision due to the smaller dynamic range of the measured longitudinal magnetization.

Existing MOLLI and SASHA techniques are limited in spatial resolution and coverage due to the need for breath‐holding, which is required to minimize respiratory motion artifacts. Typically, only a single low‐resolution 2D slice can be acquired for each breathhold with both MOLLI and SASHA. Due to the increased scan time associated with higher‐resolution 3D image acquisition of the heart, free‐breathing imaging is necessary and respiratory motion compensation imperative to ensure diagnostic image quality. Some techniques for 3D *T*_1_ mapping have already been proposed.^11^, ^12^, ^13^ However, these are based on an inversion recovery acquisition and consequently they are heart rate‐dependent. Instead, the SASHA sequence appears to be a promising candidate for volumetric free‐breathing *T*_1_ mapping of the whole heart, due to its high scan efficiency and quantification accuracy, and its robustness to heart‐rate variations which may occur over the course of a 5–10‐minute scan.

In this work, we sought to develop and assess a multishot high‐resolution 3D SASHA sequence with improved spatial resolution and coverage that can be acquired in free‐breathing with respiratory motion compensation.

## Materials and Methods

### Pulse Sequence Scheme

**Figure 1:**
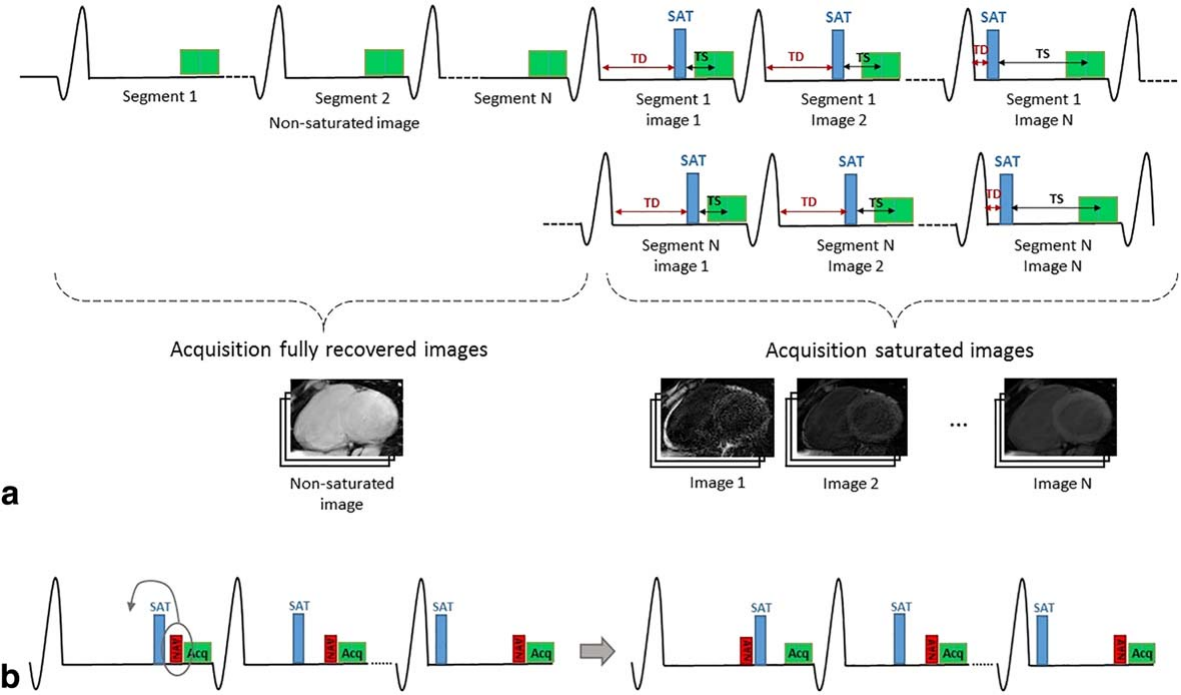
**a**: Schematic diagram of the 3D SASHA pulse sequence. First, the images without any magnetization preparation are acquired, then an interleaved acquisition is used for the saturated images, where the saturation times (TS) and the delay time (TD) is varied. **b**: For the shortest TS the position between the saturation pulse (SAT) and the navigator (NAV) is swapped.

All data were acquired on a 1.5T Ingenia MR system (Philips, Best, The Netherlands). The proposed 3D SASHA pulse sequence enables the acquisition of whole‐heart *T*_1_ mapping in free breathing. To this end, the 2D SASHA sequence^7^ was modified to make the sequence compatible with a 3D segmented *k*‐space acquisition. The sampling scheme used for 3D SASHA is shown in Fig. 1a. First, all image *k*‐space segments with no magnetization preparation were acquired, which we term “infinity image” (as we assume it is acquired after an infinite saturation delay time during data fitting). This was followed by the interleaved segmented acquisition with preceding saturation pulse and increasing saturation delays. To ensure all *k*‐space segments of the infinity image were acquired at equilibrium magnetization, “pause” cardiac cycles were added between the acquisitions of these segments. During these pauses, all RF pulses and data acquisition were switched off to allow for full *T*_1_ recovery.

To compensate for respiratory motion a 1D hemi‐diaphragmatic navigator (NAV) was used. Typically, the navigator is performed immediately before the image acquisition to allow prospective motion correction and ensure high temporal correlation between NAV and image acquisition. However, for the shortest saturation time the navigator signal may be insufficient for reliable motion estimation. Therefore, the position between the saturation pulse and the navigator was swapped for the shortest saturation time (40 msec) (Fig. 1b).

To minimize the sensitivity to B_1_ inhomogeneities the saturation pulse used for imaging was a composite saturation pulse with a train of four rectangular pulses with numerically optimized flip angles (respectively 72°, 92°, 126°, 193°), each followed by a dephasing gradient.^14^ Imaging was performed with a 32‐channel cardiac coil and the heart rate was monitored using the vector electrocardiogram (VCG) for the whole duration of the scan.

Data acquisition was performed with a segmented 3D steady‐state free‐precession (SSFP) technique and imaging parameters included: field of view (FOV) = 300 × 300 × 90 mm^3^, repetition time / echo time (TR/TE) = 3.2/1.6 msec, image resolution = 1.4 × 1.4 mm^2^, slice thickness = 8 mm, flip angle (FA) = 35°, parallel imaging with SENSE factor of 2 in phase‐encoding direction, 10 start‐up echoes, Cartesian acquisition with radial *k*‐space shutters, slice‐selective RF pulse. Thirty segments of the *k*‐space were acquired per heartbeat with a low‐high *k*‐space ordering and an acquisition window duration of 90 msec.

### Simulations

The proposed segmented multishot SASHA pulse sequence was simulated using the Bloch equations in MatLab R2014a (MathWorks, Natick, MA). The simulations were performed to analyze the effect of the added “pause” cardiac cycles between acquisitions of the infinity images and to investigate the optimal number of pauses for a given heart rate to ensure complete longitudinal magnetization recovery. The simulations for an SSFP sequence were performed for heart‐rates between 60–100 bpm, *T*_1_ of 1100 msec (precontrast myocardial *T*_1_ at 1.5T), *T*_2_ of 45 msec, 40 RF excitations per segment, FA of 35°, TR of 2.8 msec, and for different numbers of “pause” cardiac cycles.

### Phantom Experiments

A phantom with nine agar/NiCl_2_ vials was used for imaging, with *T*_1_ values ranging from 250 msec to 1500 msec,^15^ to compare *T*_1_ measurements between the gold standard inversion‐recovery spin echo (IRSE) technique, 2D MOLLI, 2D SASHA, and the proposed 3D SASHA. The IRSE pulse sequence was performed with 10 inversion times varying from 100–2000 msec. The reference *T*_1_ measurements were compared to 2D MOLLI, 2D SASHA, and the proposed 3D SASHA, in order to validate the imaging technique. Sequence parameters for the spin echo sequence were: FOV = 200 × 200 mm^2^, image resolution = 3.1 × 3.1 mm^2^, 10 mm slice thickness, TR = 8000 msec, and TE = 5.9 msec.

The MOLLI sequence was acquired with the following parameters: FOV = 300 × 280 mm, TR/TE = 2.6/1.3, image resolution = 1.7 × 2.1 mm, slice thickness = 10 mm, FA = 35°, scan time of about 12 seconds. The acquisition parameters for the 2D SASHA sequence were: FOV = 300 × 280 mm^2^, TR/TE = 2.6/1.3, image resolution = 1.7 × 2.1 mm^2^, slice thickness = 10 mm, FA = 70°, 100–700 msec saturation time (TS) for a heart rate of 60 bpm, parallel imaging with SENSE factor of 2 in the phase‐encoding direction. For the 3D SASHA sequence the saturation times (TS) were 100–700 msec for a heart rate of 60 bpm. All three imaging techniques were reconstructed to the same in‐plane resolution of 1.25 × 1.25 mm^2^.

The proposed 3D SASHA sequence was acquired with fewer *T*_1_‐weighted images along the Mz recovery curve compared to the conventional 2D SASHA sequence (9 instead of 11, including the “infinity” image) in order to keep the total scan time within 5 minutes.

All sequences were triggered with a simulated heart rate of 60 bpm and signal reception was performed with a 32‐channel cardiac coil

To validate the findings of the simulations and evaluate the optimal flip angle and number of “pause” heart cycles added between acquisitions of the fully recovered “infinity” images, the phantom was imaged using the 3D SASHA sequence with a different number of pauses and by changing the FA within the range from 25° to 85° with 10° increments. This experiment was repeated for 3D SASHA with 0, 1, 2, and 3 pause cycles for the infinity image.

### In Vivo Studies

The study was performed in accordance with the Declaration of Helsinki (2000). All subjects involved in this study provided written informed consent with study approval from the Institutional Review Board (15/NS/0030). Ten healthy volunteers without a history of heart disease were imaged using 2D MOLLI, 2D SASHA, and 3D SASHA sequences. The same imaging parameters used for the phantom experiment were employed for the in vivo study. The acquisition of the 3D SASHA sequence was performed in free‐breathing with a nominal scan duration of 4:14 (min:sec) for a heart rate of 60 bpm and 100% scan efficiency. A 1D diaphragmatic navigator was used for respiratory motion compensation with a gating window of 5 mm and a tracking factor of 0.6.

Following the validation with Bloch simulations and phantom studies, three “pause” cardiac cycles were used for all in vivo imaging.

### Image Analysis

The *T*_1_ maps were reconstructed offline using MatLab. The *T*_1_ values were calculated by fitting the image signal intensities to a three‐parameter exponential recovery curve using the fitting procedure proposed by Barral et al.^16^ Regions of interest (ROIs) were manually drawn on the phantom images and in the myocardium and the measured average *T*_1_ values were expressed as mean ± standard deviation.

Accuracy and precision are the two criterions used to evaluate the efficiency of quantitative *T*_1_ mapping.^17^ For the phantom experiments the accuracy of the 3D SASHA *T*_1_ maps was evaluated by comparing the mean *T*_1_ values with the reference measurements from the IRSE experiment using a Kruskal–Wallis test. A Bland–Altman analysis was performed to compare the different techniques and to evaluate the agreement between them. For in vivo studies the myocardial *T*_1_ values were compared with the values measured with the 2D SASHA and 2D MOLLI sequences.

The standard deviation of the *T*_1_ measurements was used to quantify the precision, ie, the spatial variability of T1 across the selected ROI, of each imaging technique.^10^

Sharpness of the myocardial borders was used as a criterion to quantify the performance of motion correction and was measured using dedicated software. The sharpness was calculated as a percentage of the steepness of the edges of the myocardium, where 100% corresponds to an ideal step edge.^18^ Measurements were done on the myocardial septum for both the 2D SASHA and the 3D SASHA *T*_1_ maps. Two blinded expert reviewers (Reviewer 1 with more than 10 years and Reviewer 2 with 5 years of experience in cardiovascular MRI) scored the *T*_1_ maps of all volunteers based on image quality, which includes both myocardial sharpness and signal homogeneity. The scale for scoring the image quality was: 1 poor, 2 average, 2 good, 3 very good, and 5 excellent image quality. The Wilcoxon rank *t*‐test was used to evaluate the statistical difference between 2D SASHA and 3D SASHA. The median was calculated and presented together with the 75% and 25% of the median itself.^19^

The American Heart Association (AHA) 17‐segment^20^ models for the 2D SASHA and 3D SASHA techniques were used to compare the two techniques and to provide a 3D visualization of the left ventricle. The myocardial *T*_1_ values of the whole volume were measured for 16 AHA segments (excluding the apical segment 17, not included in short axis view as per clinical standard) in three slices (apex, mid, base). Each segment of the final model was calculated as the average of the same segment between all the volunteers.

For statistical analysis, GraphPad Prism v. 5 for Windows (GraphPad Software, La Jolla, CA) was used and *P* < 0.05 was used to define the statistical significance.

## Results

### Simulations

**Figure 2:**
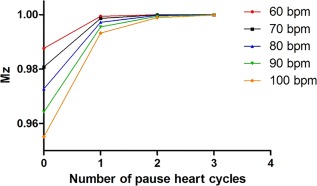
The graph shows the recovered longitudinal magnetization Mz (y‐axis) as a function of different number of “pause” cardiac cycles (x‐axis) between the acquisitions of the images without any magnetization preparation, for different heart rates.

Figure 2 shows the steady‐state M_z_ magnetization for a tissue with *T*_1_ similar to healthy myocardium on 1.5T (*T*_1_ = 1100 msec) during the acquisition of the infinity image as a function of different numbers of “pause” cardiac cycles (from zero to three). The recovery of M_z_ depends both on the number of pauses added between the infinity images and on the heart rate. M_z_ returns to the equilibrium magnetization after two pause cardiac cycles for heart rates lower or equal than 60 bpm. However, three pauses are more robust for higher heart rates, and may ensure full recovery of M_z_ even in the presence of heart‐rate variability, which can occur during a 5–10‐minute scan. Thus three pauses were chosen for all in vivo studies.

### Phantom Experiments

**Figure 3:**
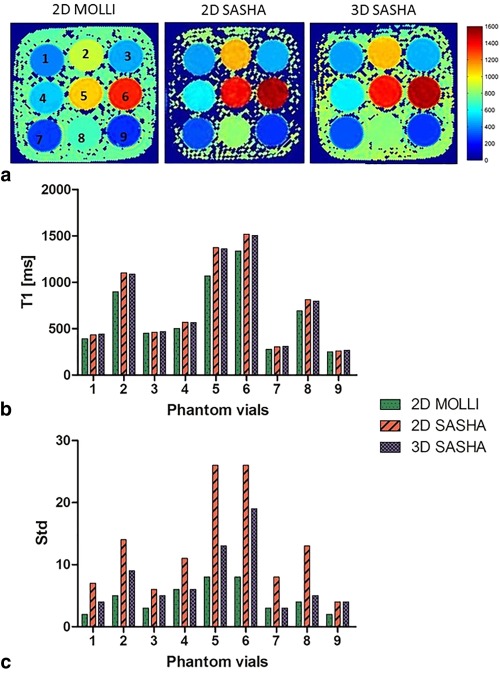
**a**: *T*_1_ map of the phantom obtained using the 2D MOLLI, 2D SASHA, and 3D SASHA sequences. **b,c**: Mean and standard deviation of the measured *T*_1_ values for all the nine vials.

**Figure 4:**
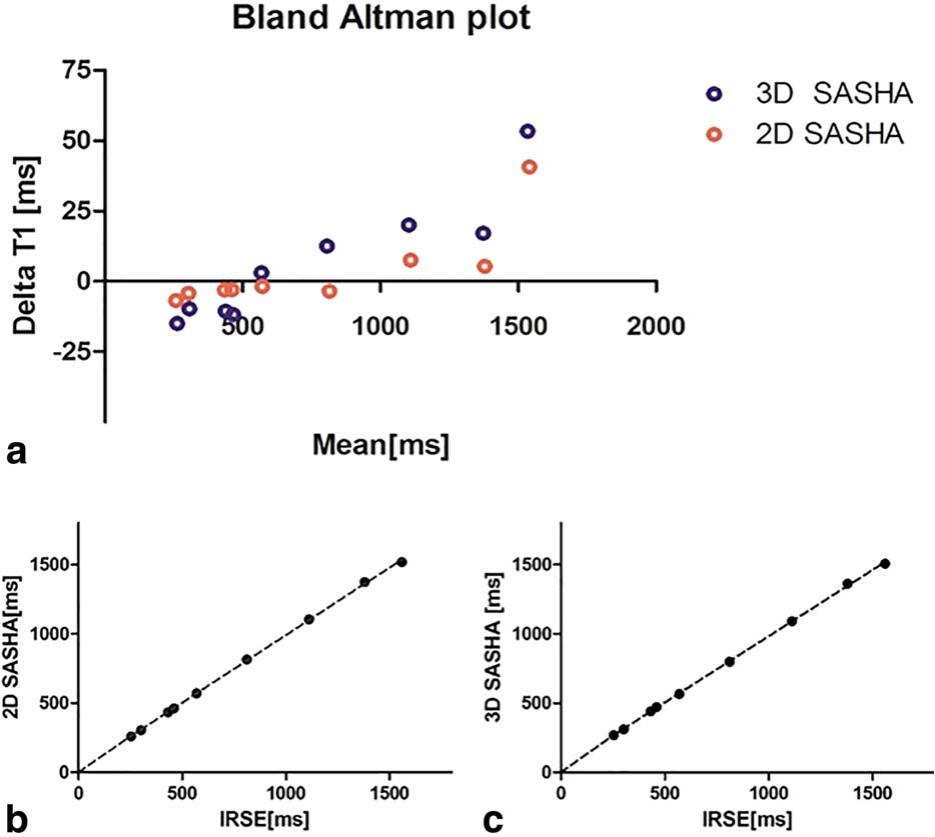
**a**: Bland–Altman analysis between IRSE and 2D SASHA (in red), and 3D SASHA (in blue) sequences. **b,c**: Agreement between IRSE and 2D SASHA and 3D SASHA sequences.

Table 1 summarizes the mean values and standard deviations calculated with IRSE, 2D MOLLI, 2D SASHA, and 3D SASHA sequences. An ROI was selected for each of the nine vials of the phantom and the average *T*_1_ value calculated. The 3D SASHA *T*_1_ map appears more homogeneous and less noisy compared to the 2D SASHA *T*_1_ map (Fig. 3a). 3D SASHA and 2D SASHA were found to have similar accuracy, while 2D MOLLI generally underestimates the *T*_1_ values. Although fewer *T*_1_‐weighted images were used for the 3D SASHA compared to 2D SASHA (9 for 3D SASHA vs. 11 for 2D SASHA) the precision of the 3D SASHA is higher, as measured by the lower standard deviation (Fig. 3b‐c). The Bland–Altman analysis in Fig. 4a compares the reference *T*_1_ values measured with the IRSE sequence with the *T*_1_ values measured respectively with 3D SASHA and 2D SASHA sequences. For the 3D SASHA, the mean difference is 6.6 msec and the limits of 95% agreement are –36.6msec and 49.8 msec, while for the 2D SASHA the mean difference is 3.5 msec and the limits of 95% agreement are –25.3 msec and 32.3 msec. The 3D SASHA *T*_1_ values are in good agreement with the 2D SASHA *T*_1_ values with significant correlation (*P* < 0.0001, *r* = 0.9998). The estimated *T*_1_ values using the 3D SASHA and the 2D SASHA sequences correlate extremely well (*r* = 0.9997) with the IRSE sequence (Fig. 4b,c). The agreement in *T*_1_ values between the 3D SASHA and IRSE was confirmed by the Kruskal–Wallis test (*P* = 0.9947). The *T*_1_ estimation for the vial with the highest *T*_1_ value deviated from the expected line of identity due to an insufficient recovery time between successive inversion pulses used for the IRSE sequence. This led to a wrong estimation of the highest *T*_1_ values with the IRSE sequence and consequently to a mismatch between the 3D SASHA and the IRSE *T*_1_ value for the vial with the highest *T*_1_.

**Table 1:** Mean and Standard Deviation for All the Phantom Vials, Respectively for Spin‐Echo, 2D MOLLI, 2D SASHA, and 3D SASHA Sequences

Phantom vial	Reference T1 [msec]	Spin‐echo T1 [msec]	2D MOLLI T1 [msec]	2D SASHA T1 [msec]	3D SASHA T1 [msec]
1	430	431.8 ± 10	391 ± 2	434.9 ± 7	442.4 ± 4
2 (Myocardial‐like)	1090	1111.6 ± 31	898.4 ± 5	1104 ± 14	1091.5 ± 9
3	458	459.4 ± 9	451.8 ± 3	462.4 ± 6	471.2 ± 5
4	562	569.3 ± 13	503.5 ± 6	571.1 ± 11	566.2 ± 6
5	1333	1379.7 ± 55	1063.5 ± 8	1374.3 ± 26	1362.6 ± 13
6 (Blood‐like)	1489	1559.8 ± 62	1338 ± 8	1519.1 ± 26	1506.4 ± 19
7	300	301 ± 7	277.3 ± 3	305.2 ± 8	310.7 ± 3
8	803	811.6 ± 17	692.2 ± 4	815.1 ± 13	799.1 ± 5
9	255	253.9 ± 6	252.4 ± 2	260.7 ± 4	268.8 ± 4

**Figure 5:**
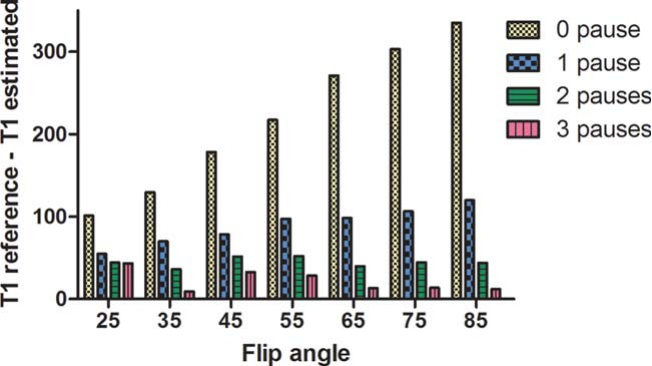
The graph shows the difference between the gold standard *T*_1_ and the measured *T*_1_ for vial number 2 (reference *T*_1_ = 1090 msec), using different combinations of number of pauses between the acquisition of the “infinity” images (from 0 to 3) and flip angles (from 25° to 85°).

Figure 5 shows the results obtained from the phantom experiment using different combinations of FAs and number of “pause” heart cycles; only the results from the vial with myocardial‐like *T*_1_ are illustrated. The graph shows the difference between the reference *T*_1_ value and the measured *T*_1_ values, and the best agreement was obtained using an FA of 35° and three “pause” heart cycles, which confirms the results obtained from the simulations.

### In Vivo Studies

**Figure 6:**
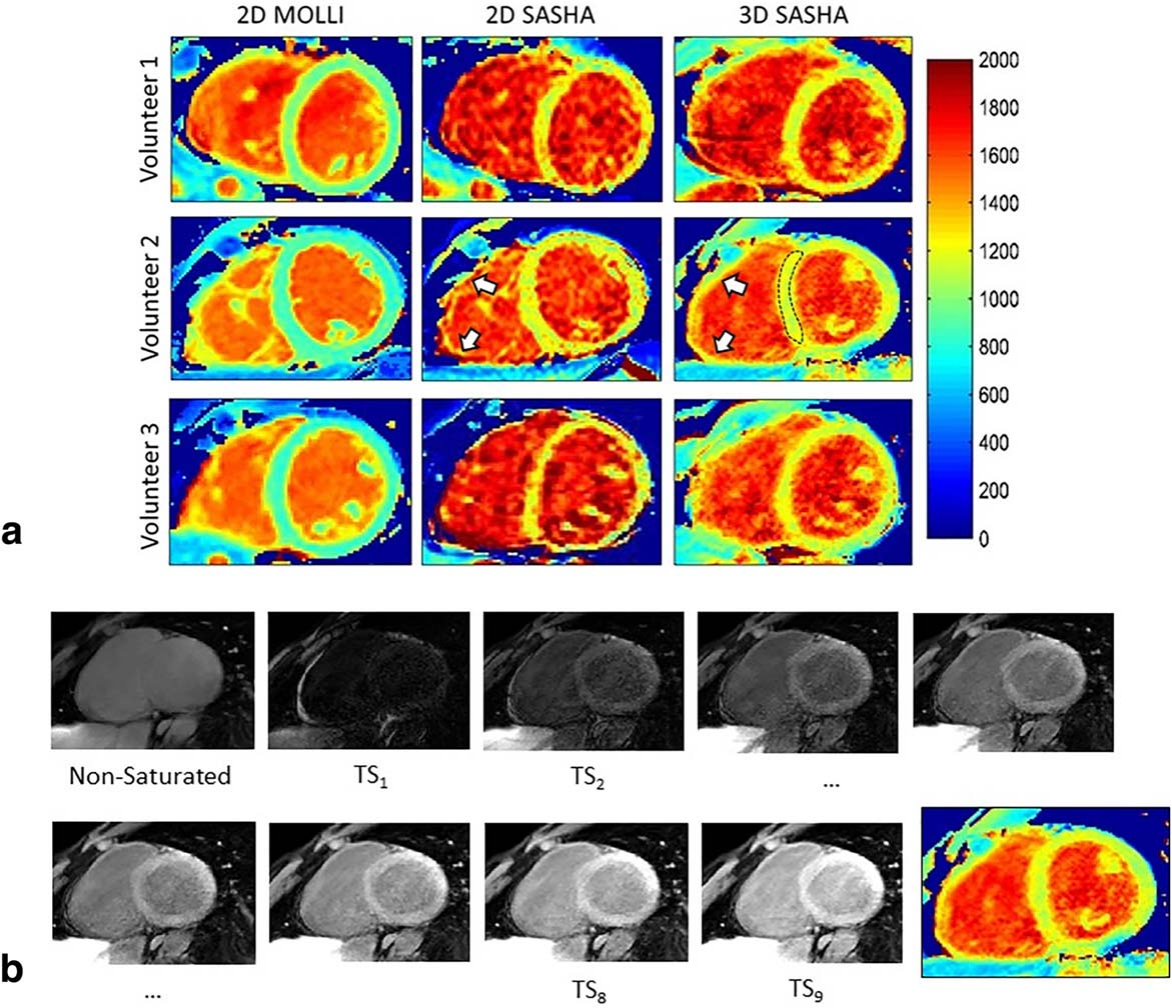
**a**: Mid‐ventricular myocardial *T*_1_ maps of three healthy subjects using the sequences 2D MOLLI, 2D SASHA, and 3D SASHA sequences. The average *T*_1_ values were calculated by manually drawing an ROI in the septum of the myocardium. The higher resolution of the 3D SASHA technique allows to better delineate the right ventricle (white arrows). **b**: Nine images acquired at different saturation times for Volunteer 2.

**Figure 7:**
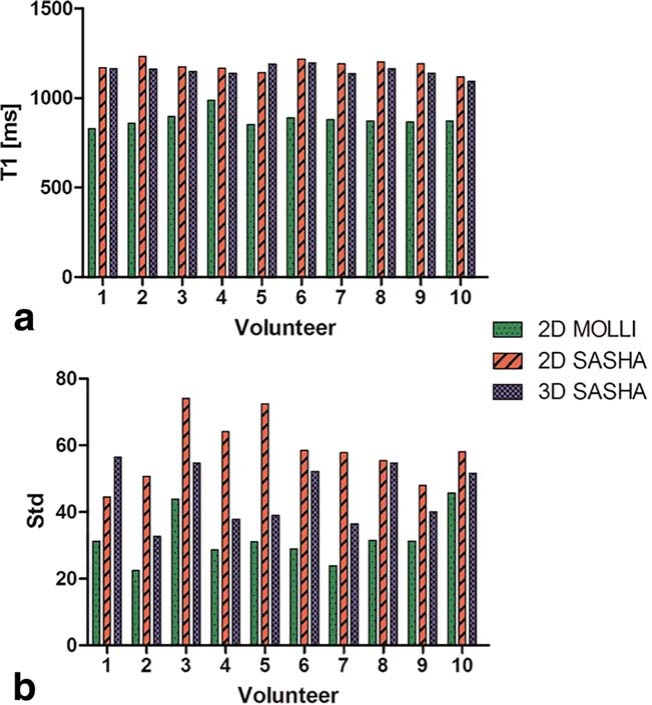
Mean and standard deviation of the myocardial *T*_1_ measurements of the 10 healthy subjects using 2D MOLLI, 2D SASHA, and 3D SASHA sequences.

Figure 6a shows the myocardial *T*_1_ maps of three volunteers obtained with the 2D MOLLI, 2D SASHA, and 3D SASHA sequences. The single *T*_1_‐weighted images acquired for Volunteer 2 are shown in Fig. 6b. For all volunteers there was good agreement between the myocardial *T*_1_ values measured with the 2D SASHA and the 3D SASHA sequences, while the *T*_1_ values obtained with the 2D MOLLI sequence were considerably lower (Fig. 7a). A trend of improvement in terms of precision was visible with the proposed imaging technique compared with the 2D SASHA (Fig. 7b). The average *T*_1_ values of all subjects for the 2D MOLLI, 2D SASHA, and 3D SASHA sequences were 881 ± 32 msec, 1181.2 ± 32msec, and 1153.6 ± 28 msec, respectively. The duration of the 3D SASHA scan was of 12:1 ± 1:3 (min:sec) with a scan efficiency of 32%.

The average myocardial border sharpness measured on the 2D SASHA *T*_1_ maps was significantly higher compared to 3D SASHA *T*_1_ maps (respectively 26% ± 2 and 18% ± 3).

The agreement in image quality between the observers was 80% for the 2D MOLLI *T*_1_ maps, and 50% for the 2D SASHA and 3D SASHA *T*_1_ maps. In general, we observed better image quality for the 2D MOLLI *T*_1_ maps (median 5, 75% of the median of 5, 25% of the median of 4), with significant statistical difference with both 2D and 3D SASHA (respectively *P* = 0.0053 and *P* = 0.0047). There was no statistical difference between 2D SASHA (median 2, 75% of the median = 3, 25% of the median = 2) and 3D SASHA (median 3, 75% of the median = 3, 25% of the median = 2).

**Figure 8:**
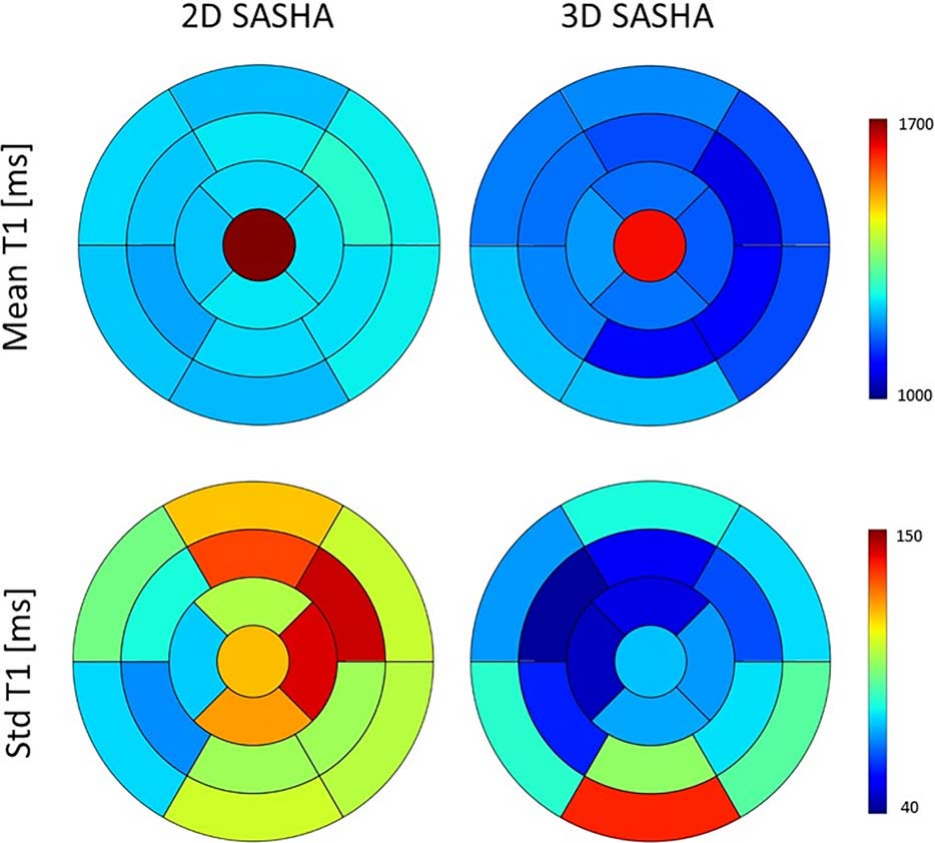
Bull's eye plots of the left ventricle. Mean *T*_1_ values (top row) and standard deviations of the *T*_1_ measured (bottom row) are shown for both 2D SASHA and 3D SASHA sequences. Each segment is the average between 10 volunteers and contains the average myocardial *T*_1_. The red central segment represents the blood.

Figure 8 shows the bull's eye plots of the left ventricle for the 2D SASHA and 3D SASHA imaging sequences. The AHA model was calculated for both the mean and standard deviation of the *T*_1_ values measured across the left ventricle. The average myocardial *T*_1_ values are homogeneous across the left ventricle for both of the imaging techniques. The standard deviation for the 3D SASHA is lower compared to the 2D SASHA, which could be explained by the theoretical higher signal‐to‐noise ratio (SNR) for the 3D technique. However, a higher standard deviation was observed in the inferior wall of the basal slice, which could be explained by slice profile imperfection and motion artifacts.

## Discussion

In this study we proposed and validated a free‐breathing 3D SASHA sequence for *T*_1_ mapping of the whole left ventricle. The sequence was validated with simulations, phantom, and in vivo experiments.

The proposed 3D *T*_1_ mapping approach was based on the well‐established 2D SASHA pulse sequence design.^7^ The main difference between 2D SASHA and the proposed 3D sequence was that segmented *k*‐space acquisition was required to enable high‐resolution 3D acquisition. A Cartesian acquisition with radial *k*‐space shutter was used, which is commonly used for magnetization‐prepared heart imaging. However, no further analysis was conducted to investigate the effect of the acquisition scheme.

The in vivo studies confirmed the phantom measurements in terms of accuracy between 2D SASHA and 3D SASHA sequences, as similar average myocardial *T*_1_ values were obtained. The standard deviations of the 3D *T*_1_ measurements were lower compared to the 2D SASHA. Improved subjective image quality with 3D SASHA over 2D SASHA was observed, although this finding was not significant. This could be attributed to the fact that a 3D imaging sequence inherently benefits from higher SNR compared to a 2D method.

As demonstrated in the phantom experiments, the proposed 3D *T*_1_ mapping imaging sequence improves accuracy compared to 2D MOLLI and precision compared to 2D SASHA. This is a promising result and warrants further evaluation of the 3D SASHA imaging technique in clinical practice. Also, a free‐breathing *T*_1_ mapping approach would be useful in patients who cannot hold their breath due to respiratory problems.

Other 3D *T*_1_ mapping techniques have already been proposed,^11^, ^12^, ^13^ which use an inversion recovery pulse to generate *T*_1_ weighting. The proposed 3D SASHA technique uses a saturation recovery pulse, which has the advantage of completely erasing the history of the longitudinal magnetization between the acquisitions, thus reducing the heart rate dependency as well as improving scan efficiency. Further work will investigate other available *T*_1_ mapping techniques with the proposed 3D SASHA sequence. For the 3D SASHA imaging techniques, an incomplete and incorrect saturation could be an important source of errors in the final *T*_1_ map. In the proposed 3D SASHA sequence a composite saturation pulse was used, with a saturation efficiency of 0.989 ± 0.005, estimated from the exponential fitting curve.^7^ The high saturation efficiency confirms the results reported from previous studies.^7^ The sharpness of the septum was significantly lower with the 3D SASHA compared to the 2D SASHA *T*_1_ map. This is likely due to residual respiratory motion artifacts. Improved respiratory motion compensation strategies, such as self‐navigation^21^ or image‐based navigation,^22^, ^23^, ^24^ may improve image sharpness while simultaneously helping to reduce scan time. Nevertheless, the visual score test showed a trend of improved image quality for the 3D SASHA compared to the 2D SASHA *T*_1_ maps. In general, better image quality and better visualization of the myocardial borders would be beneficial where trasmurality of scar should be assessed. In addition, high‐resolution *T*_1_ mapping may be especially beneficial for fibrosis quantification in the thin wall of the right ventricle. The AHA model showed in general a good homogeneity of the myocardial *T*_1_ values measured across the left ventricle. However, *T*_1_ values in the lateral wall of the mid ventricle were somewhat lower compared to other segments. This is consistent with previous findings showing lower *T*_1_ values in this region, which is likely due to susceptibility artifacts caused by the air–myocardium interface.^25^

In this work a 3‐3‐5 MOLLI scheme was used, which was the clinically used *T*_1_ mapping technique at the time of the study. Recently, the 5‐3 MOLLI scheme has become more popular, due to the shorter breath‐hold duration. Further work is required to compare the precision, accuracy, and clinical merits of more recent *T*_1_ mapping techniques with the proposed 3D SASHA.^9^

The proposed 3D SASHA imaging sequence has been extensively tested and validated with simulations, phantom studies, and in vivo experiments on healthy volunteers. Further work is required to evaluate this technique in patients with myocardial scar, as well as for postcontrast *T*_1_ mapping.

Image resolution of the 3D SASHA sequence is mainly limited by the scan time, which is ∼12 minutes for the current spatial resolution of 1.4 × 1.4 × 8 mm^3^. The implementation of novel motion compensation techniques, such as image‐based navigation, correcting for both translational and nonrigid motion,^22^ removes the requirement for gating and allows using all acquired data for image reconstruction, thereby significantly reducing scan time or increasing spatial resolution. In addition, improved motion compensation may also improve myocardial sharpness and overall image quality. The use of compressed sensing reconstruction could also further accelerate imaging time.^26^

An additional step of retrospective image registration^27^ or removal of severely motion corrupted images could be beneficial to correct for residual motion in the *T*_1_ map, which could further improve the reliability of the *T*_1_ measurements.

Scan time is also affected by the number of pauses added between the *k*‐space segments of the infinity images. Reduction of the number of pauses could be achieved by introducing some subject‐specific correction in the fitting step. A reduced number of images acquired with the 3D SASHA sequence could be another possible solution to shorten the acquisition time, although the trade‐off between quantification precision and total acquisition time will have to be investigated further.

In conclusion, the proposed 3D SASHA *T*_1_ mapping technique allows acquiring high‐resolution myocardial *T*_1_ maps of the whole left ventricle completely in free breathing with good accuracy. While the accuracy is in the range of the 2D SASHA sequence, the precision is improved.

## Supporting information

Additional supporting information may be found in the online version of this article

Supporting Information
