# Supplementary figures and images for: 3D myocardial T 1 mapping using saturation recovery
Source: J Magn Reson Imaging. 2017 Feb 2;46(1):218–27. doi: 10.1002/jmri.25575 (PMC5518207; doi:10.1002/jmri.25575)

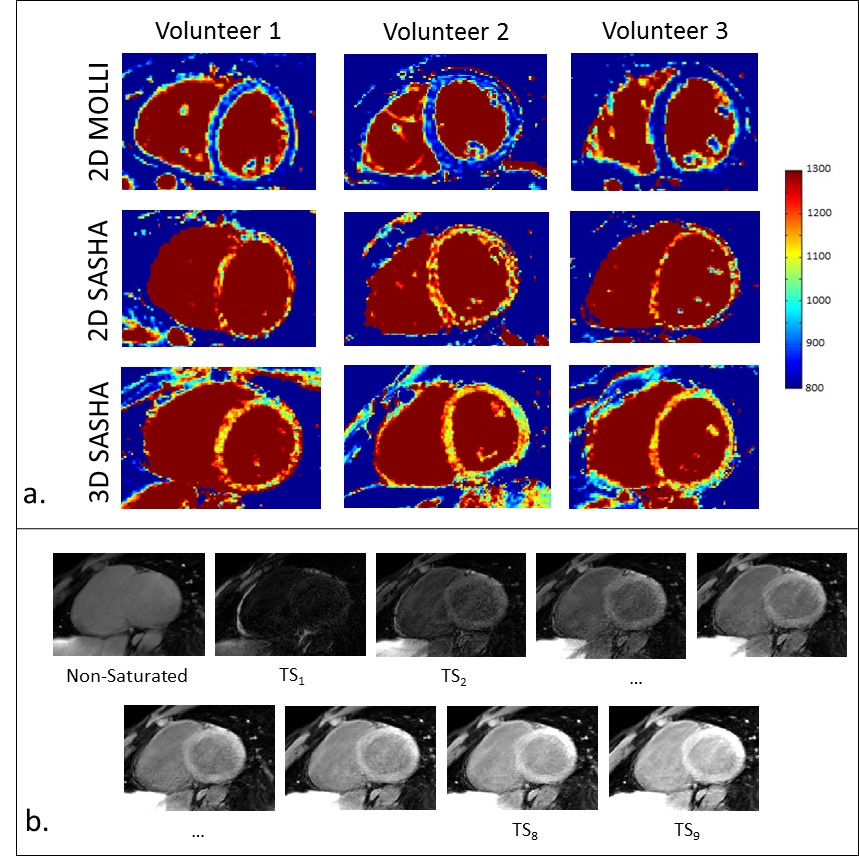

Supplement: Supplementary file 1 — Supporting Information [file JMRI-46-218-s001.tif]
